# How to Keep Sustainable Development Between Enterprises and Employees? Evaluating the Impact of Person–Organization Fit and Person–Job Fit on Innovative Behavior

**DOI:** 10.3389/fpsyg.2021.653534

**Published:** 2021-04-30

**Authors:** Yuan Tang, Yun-Fei Shao, Yi-Jun Chen, Yin Ma

**Affiliations:** ^1^School of Management, Sichuan University of Science and Engineering, Zigong, China; ^2^School of Management and Economics, University of Electronic Science and Technology of China, Chengdu, China; ^3^School of Philosophy and Sociology, Lanzhou University, Lanzhou, China

**Keywords:** person-organization fit, person-job fit, innovative behavior, resilience, partial least squares, high tech-industries, turnover intention

## Abstract

High-tech industries often regard workers as their main source of value creation. In order to stimulate their employees' willingness to innovate and their innovative behavior and reduce the turnover intention, companies are now seeking to establish employer–employee relationships in which their employee's willingness to stay is not simply driven by extrinsic motivations. Therefore, it is an important topic in human resources for companies to implement measures that encourage employees to willingly devote themselves to their jobs and consider organizational growth as a component of their career development. This study aimed to investigate the effect of person–organization fit and person–job fit on employees' innovative behavior and turnover intention *via* the mediators including job satisfaction and organizational commitment. Six hundred ninety-seven employees from China's eight major high-tech industries were examined in this study, and the empirical results were analyzed using partial least squares. Based on the results, it is suggested that the person–organization fit and person–job fit are both crucial factors affecting employees' job satisfaction and organizational commitment, which, in turn, increase employees' willingness to innovate in their jobs and reduce their turnover intentions. Furthermore, this study could serve as a reference for companies in selecting employees, promoting job satisfaction, and developing strategies for sustainable development.

## Introduction

Based on the current condition of the global economy, organizations are required to rapidly respond to changes in the business environment, make timely changes in strategies, and adapt to various circumstances in order to survive. They need to deal with challenges that are internal and external by raising resilience at the organizational level (Ramdani et al., 2020). As organizational adaptability mainly depends on employee responses to change, organizations also need to focus on the behaviors and attitudes of their members. This is especially the case in the current time, when the COVID-19 (coronavirus disease 2019) pandemic caused a serious economic recession in most sectors and a large number of employees were thus laid off (Heinonen and Strandvik, 2020).

In the traditional recruitment process, talents were mostly recruited based on the duties and qualifications specified in job descriptions, i.e., on person–job (PJ) fit, which assumes that individuals with sufficient knowledge, skills, and abilities would be competent in handling their job tasks, duties, and responsibilities. This PJ fit–based recruitment model, however, fails to consider the fit between employees' personal attributes or values and organizational culture or management concepts, as well as the possibility of employees resigning if they are unable to adapt to their organization. Frequent personnel turnover not only requires massive human and material resources from organizations to replace talents, but also negatively impacts organizational atmosphere and employee morale, which, in turn, hinders organizational development. In practice, companies search for qualified employees, while employees seek to join suitable companies. Therefore, the selection of suitable employees should be based not only on the compatibility between their professional skills and the job requirements, as it is also important to consider person–organization (PO) culture fit and PO value fit. The traditional PJ fit–based human resource management system can no longer meet the requirements for organizational development.

In the past decade, the concepts of congruence and fit have received wide interest in organizational research (Amos and Weathington, 2008). Prior studies have focused on its linkage with job satisfaction and team performance (e.g., Saks and Ashforth, 1997; Verquer et al., 2003; Hoffman and Woehr, 2006; Piasentin and Chapman, 2007; Vilela et al., 2008; Kammerhoff et al., 2019). Recently, various researchers have increasingly turned their attention to the compatibility between an individual and their working environment, instead of focusing only on the fit between an individual's personality traits and a career. Research results may become more objectively accurate if individual factors (such as skills, capabilities, requirements, and values) and organizational factors (such as working conditions, organizational culture, and organizational climate) are simultaneously considered. The concept of PO fit has been widely studied. At the individual and organizational levels, the interactive relationships between individual and organizational behaviors, as well as the role of PO fit in the employment process (selection, hiring decisions, career decisions, etc.) and its effect on individual and organizational results, have become important research topics. Hence, PO fit has opened up sustainable horizons for the research fields of human resources and organizational behavior.

PO fit has attracted significant attention partly due to rapid changes in job characteristics that have led to revised job descriptions, such that organizations now possess sufficient flexibility to actively adapt to various dynamic environments (Sung and Kim, 2020). At the same time, it is essential for employees to be prepared at all times to make changes to their current tasks, i.e., effortlessly adapt to different work teams or respond appropriately in uncertain and unknown situations. A sole focus on an individual's fit with a certain job fails to account for the possibility of him or her being transferred or retrained if he or she is unable to adapt to changes in his or her job content, which would force an organization to place a greater emphasis on searching for better fitting employees. Hence, PJ fit is no longer the only indicator in the recruitment process, as PO fit implemented throughout the employment and socialization processes is often used to retain employees with flexibility and organizational commitment (Kristof, 1996). It can be seen that PO fit has important significance and effects with respect to individual and organizational variables. However, some researchers have suggested that it is necessary to include PO fit and PJ fit to meet these requirements (Bowen et al., 1991).

In recent years, researchers have focused on organizational teams, and there has been a lack of empirical research on individuals. For instance, in a meta-analysis by Halfhill et al. (2005), more than half of the studies examined had University students as participants, while the rest involved the participation of professional teams. Therefore, the studies that involved professional teams mostly required these teams to complete specific tasks, whereas the studies that recruited University students generally involved only problem-solving tasks. This is because more resources (in terms of manpower, materials, funding, and time) are required to conduct the team-focused studies, which leads to a lack of empirical research and insufficient scientific evidence in this area. Therefore, this study applied partial least squares (PLS) to verify the mediating mechanism of organizational fit through job satisfaction and organizational commitment on employees' innovative behavior and willingness to leave their jobs. Through the collection and collation of relevant literature, analysis, and synthesis, some research hypotheses were derived, and finally the empirical data were analyzed. This study would contribute to provide the research findings to bridge the gaps in the related research field, as well as to provide reference for the industry in selecting team members, promoting job satisfaction, and the strategic development of sustainable development of the team.

## Theoretical Background and Literature Review

### Person–Organization Fit

As the meaning of the word “environment” differs according to various contexts, different scholars have used various constructs to explain the effects of an individual's environment on their behavior (Edwards and Shipp, 2007). The different levels of fit play important roles in employment relationships, and they each emphasize different things (Edwards and Billsberry, 2010). Kristof-Brown et al. (2005) broadly defined the compatibility fit between an individual and an organization when the characteristics of the individual (values, personality, or goals) and the features of the work environment (values, norms, or goals) are well matched (Kristof-Brown et al., 2005).

Schneider (1987) proposed the attraction–selection–attrition (ASA) model and indicated that individuals would be attracted to organizations that have values and goals that are similar to theirs. After willingly joining or after being selected to join an organization, individuals whose attributes differ greatly from those of the organization encounter conflicts within it, which drives them to make a decision on whether they should stay or leave their organization. The ASA model explains that it is the environment that is shaped by individuals and their behaviors and not the other way around. This is an extension of the concept of supplementary fit and emphasizes the importance of a supplementary relationship between individuals and their organization. However, the ASA model lacks specific descriptions as it is based on the concepts of normative theory. Even though it is able to explain the congruence of value fit, it is unable to provide thorough explanations of actual organizational operations as organizations often emphasize attributes other than individual values (such as individual capabilities) during their employment process. It is also possible that employees would rather stay with an organization even when they do not agree with its values or are uninterested in its goals.

In recent years, researchers have proposed approaches based on integrated perspectives to explain PO fit. Kristof (1996) proposed a more complete PO fit model and explained that individuals and organizations not only have basic attributes, but also have respective requirements and available resources. This integrated model incorporates various PO fit concepts and also considers the standpoints of supplementary fit and complementary fit.

### Person–Job Fit

The concept of PJ fit was first proposed by Caldwell and O'Reilly (1990), who defined it as the congruence between individual personalities and working environments, or the compatibility between individuals and specific jobs, i.e., the compatibility between the skills and needs of employees and jobs. The PJ fit stresses the level of fit between an individual's attributes or capabilities and a certain job or task (Edwards, 1991; Cable and Judge, 1996; Kristof-Brown, 2000).

After reviewing 92 PJ fit–relevant studies, Edwards (1991) proposed a PJ fit model based on demands and abilities to predict organizational outcomes. PJ fit is generated when the supply of a job satisfies employees' needs or desires, or when employees' abilities are able to meet job demands. Cable and DeRue (2002) later extended the concepts of Kristof (1996) and Edwards (1991) and clarified that the concept of PJ fit is complementary and consists of Needs-supplies (NS) fit and Demands-abilities (DA) fit.

As PO fit and PJ fit are both important topics in human resources management and organizational behavior, and as they have close relationships with work outcomes, PO fit and PJ fit were the main focuses of this study. PJ fit affects an individual's work behavior, performance, and outcomes (such as work performance, organizational identification, job satisfaction, and turnover intention) in an organization (Brkich et al., 2002). Different outcome variables can be predicted by different fit factors. Cable and DeRue (2002) verified that employees express more organizational identification when they believe that their values fit with organizational values and that the relationship between PJ fit and employees' job satisfaction is statistically significant. Edwards (1991) revealed the positive correlations between PJ fit and behavioral consequences such as job satisfaction, low work stress, performance, attendance rate, and employee retention rate. Most studies have shown the positive effects of high levels of fit, but conversely, some studies have also shown the negative effects of high levels of fit, which affects organizational adaptability and innovative capabilities.

Even though they overlap partially, the concepts of PO fit and PF fit have no relevance to each other. Researchers believe that individual experiences vary with different jobs or organizations, which result in changes in fit (O'Reilly et al., 1991). PJ fit is basic of the specific compatibility between individuals and jobs, which includes fit in capabilities, job characteristics, interests, or personalities. On the other hand, PO fit is based on the fit between values and goals of employees and organizations. Even if an individual was able to adapt to a specific task and had good PJ fit, changes in the organizational demands of employees would remain unchanged and would not be influenced by the individual's adaptability or competence. PO fit, however, varies according to changes caused by interactions between individuals and their organizational environment and attributes. Therefore, the level of interaction in PO fit is greater than that in PJ fit, and in essence. There is a certain level of difference between these two fits. Furthermore, the effectiveness of distinguishing these two fits has been supported by empirical evidence (Lauver and Kristof, 2001).

Regarding the processes of promotion and recruitment in organizations, Kristof-Brown (2000) pointed out that PJ fit had more explanatory power than PO fit. Kristof-Brown et al. (2002) examined how individuals combined their perceived PO fit, PJ fit, and PG fit while developing job attitudes. They found out that each type of fit influenced distinct impacts on job satisfaction and turnover intentions, respectively, whereas PJ fit had a significant effect on job attitudes. As it is likely for relevant variables to be discussed in studies on fits based on different perspectives, multidimensional measurements should be carried out when researching levels of fit (Westerman and Cyr, 2004). A study may be more complete if multidimensional measurement and research were simultaneously performed when examining fits.

In recent years, many studies on PO fit and PJ fit have used direct measurements of perceived fit (Cable and Judge, 1996; Netemeyer et al., 1997; Lauver and Kristof, 2001; Cable and DeRue, 2002; DeRue and Morgeson, 2007) in lieu of indirect measurements, as empirical evidence has suggested that direct measurements are more capable of explaining variance than indirect measurements (Tepeci and Bartlett, 2002; Hoffman and Woehr, 2006).

Direct measurements were adopted in this study in lieu of indirect measurements, as this approach has better explanatory power regarding the effects of an individual's level of perceived fit on their job attitudes (Kristof, 1996; Cable and Judge, 1997). Research has also shown that participants can self-assess their capabilities with a certain level of accuracy (Atwater et al., 1998), which proves that individuals are able to clearly identify different types of fit (such as PO fit, DA fit, NS fit) when they conduct measurements of perceived fit.

### Job Satisfaction

Job satisfaction is a unitary concept and refers to the overall emotional state of employees as they psychologically and physiologically face factors related to their working environment. In other words, it is formed from the individual subjective responses from employees toward their work scenarios. Robbins et al. (2015) defined job satisfaction as the joyous or positive feelings about one's job derived from one's evaluation of their work experience or job characteristics. Hence, job satisfaction is the subjective emotional response from an individual toward their job, and this feeling is influenced by various factors. These affective descriptions often grow during the process of evaluating an individual's work experience. On these bases, job satisfaction is one's affective response toward their job. Robbins et al. (2015) suggested that job satisfaction not only can be defined as an affective component, but also may be developed into a cognitive component that can be obtained by evaluating job conditions, opportunities, and supply. According to this definition, cognitive job satisfaction includes the process of making comparisons. Therefore, job satisfaction is a relative concept as comparisons can be made based on reference values during appraisals and not simply based on emotional judgments.

Job satisfaction is a term often used around the workplace and is often discussed in the fields of psychology and management studies. Definitions of job satisfaction vary according to different fields of research. The job attitude of an employee that has positive and joyful feelings toward their job can be defined as job satisfaction, whereas the opposite can be defined as job dissatisfaction. Another approach for defining job satisfaction is to compare the gap between employees' expected and actual rewards. Job satisfaction can be viewed as an individual's general attitude toward their job (Robbins, 2005); it represents the level to which workers like or dislike their jobs. Employees reflect their feelings by expressing satisfaction and positive attitudes toward their jobs and organizations.

### Organizational Commitment

Organizational commitment is the degree of personal identification with and commitment to a particular organization, which enables members of the organization to internalize the goals of the organization and display behavior beneficial to the organization (Mowday et al., 1982; Naz et al., 2020). Podsakoff et al. (2000) argue that employees will demonstrate organizational citizenship behavior as a reward for organizational support. Staw and Salancik (1982) proposed that organizational commitment allows members to be willing to strive for the organization regardless of the outcomes of their actions. Employees with higher retention commitments are more devoted to their jobs. Deluga (1994) pointed out that individuals with organizational commitment receive similar rewards from their supervisors or organizations, which stimulates their behavior to perform practical actions that contribute to their organizations.

Organizational commitment is an element of employees' work behavior within an organization, an attitude or orientation that links or attaches individuals to the organization as a whole. When employees identify with the organization and its goals and want to be part of the organization, organizational commitment is inversely related to turnover and absenteeism rates; i.e., organizational commitment is an emotion of affiliation, identification, and participation (Robbins, 2005). In other words, when members are highly committed to the organization, they can bring a high degree of centripetal force and competitiveness to the organization, which in turn can create insecurity or turnover risk.

### Employee Innovative Behavior

Innovation is a major source of competitive advantages for today's organizations (Drucker, 1999). Employee innovative behavior helps to bring new and feasible solutions and ideas to enterprise related services, products, and business processes. The starting point of organizational innovation is when people demonstrate innovative behaviors in their work, including using creativity, identifying problems, making the most of opportunities, and actively thinking of and then implementing ideas to launch new services, products, and even new markets. Therefore, whether it is to inspire members to develop their creativity or to motivate them to implement their creativity is a subject of concern to scholars of organizational innovation (Yuan and Woodman, 2010; Anderson et al., 2014).

Janssen (2000) stated that employee innovative behavior refers to the generation, implementation, and application of new thoughts in a group or organization. The innovative ideas of employees are important to the organization, not only to increase work efficiency, but also to enhance the performance of the organization (Baer and Frese, 2003). Therefore, how organizations motivate their employees to innovate and create the right environment for them to do so and whether they can support and assist their employees to execute innovative ideas have been the critical theme on the research field of organizational behavior (Tierney and Farmer, 2002).

The impact of organizational social context on workers' innovative behavior is through the members' self-cognition process (Yuan and Woodman, 2010). Employees' self-confidence or sense of innovation are the important roles about efficiency in completing innovative tasks (Tierney and Farmer, 2002). Nowadays, enterprises are facing the dilemma of rapid technological change and harsh business environment. In this study, “employee innovative behavior” is defined as the overall behavioral process of employees' search for, establishment of, execution of, and successful implementation of ideas for new technologies, new processes, new techniques, or new products to turn them into useful products or services.

### Turnover Intention

According to social exchange theory, employees who are cared for by their organizations will reciprocate by performing actions that benefit their organizations (Blau, 2017). In contrast, employees will reduce their organizational trust and commitment if they perceive that their organization has lost faith in them. In the case of repatriates, those who perceive that their parent company has failed to fulfill the psychological contracts and commitments that were established between the two sides would no longer express loyalty or perform mutually beneficial actions for their parent company, nor would they regard their parent company as a working environment filled with support and remain in their positions (Feldman and Thomas, 1992). Therefore, it is inferred that when repatriates perceive that their parent company has lost faith in them, they will feel dejected as the gap between their expectations and reality enlarges (Feldman et al., 2000). As they are dissatisfied with the outcomes after being repatriated (Suutari and Brewster, 2003; Vidal et al., 2007), they would proactively look for other job opportunities and develop higher turnover intentions. An employee with a turnover intention could bring about an operational crisis for his or her company (Karsh et al., 2005).

Based on the turnover intention model proposed by Szilagyi (1979), job satisfaction is an important antecedent variable for turnover intention or behavior. This researcher also believed that the level of job satisfaction can negatively impact turnover intention or behavior. In the subsequent turnover models proposed by Bluedorn (1982) and Michael and Spector (1982), organizational commitment was also considered as an important antecedent variable affecting turnover intention, in addition to job satisfaction, as employees with low organizational commitment may leave their organizations. Karsh et al. (2005) determined that perceived desirable working environments and organizational conditions positively affect workers' commitment and satisfaction, as low commitment and satisfaction negatively affect turnover intention. On the other hand, if a company fulfills its psychological contracts with and commitments to repatriates, or perhaps even performs beyond these expectations, these repatriates would strongly perceive that their parent company supports and cares about them and is worthy of their trust. Based on the principles of a mutually beneficially exchange, these repatriates would display high levels of positive affection and loyalty toward their parent company and would perceive that it would be a huge loss for both parties if they decided to leave a company worthy of their trust.

## Research Methodology

### Research Hypotheses Development and Research Framework

Past studies had shown that PO fit affects an individual's preference for organization, commitment to work, and performance (Piasentin and Chapman, 2007; Anderson et al., 2008; Clercq et al., 2008). One of the reasons why employees leave the company is because individuals and organizations do not fit together (Gooley, 2001). When personal values are consistent with organizational values and their resilience is better, teams and organizations would have a higher level of fit and employee satisfaction with a greater willingness to stay in the organization (Chatman, 1989). Conversely, the incompatibility between the individual and the perception of the work will make the employee consciously have a poor degree of fit between the work and the actual work and thus have a sense of job frustration and poor job satisfaction and have a negative impact on personal health (Caplan, 1987; Dooley, 2003). In addition, personal and work perceptions do not fit and may allow employees to leave their current jobs to better suit their talents (Bretz and Judge, 1994; Feldman et al., 2002). Therefore, the perception of the individual and the job adaptation assessment are not suitable, which will negatively affect the worker's job satisfaction (Livingstone et al., 1997; Cable and DeRue, 2002).

In addition, Huang and Hsiao (2007) found that the advantageous working conditions would influence the job satisfaction and organizational commitment positively. A higher employee-to-organization fit could also result in better organizational commitment and job satisfaction (Vancouver and Schmitt, 1991; Verquer et al., 2003; Kristof-Brown et al., 2005). It is also possible that employees are unable to understand the company's literacy or integration into the organization and that employees have a lack of ownership of the organization, so employees tend to choose to leave (Autry and Daugherty, 2003). Cable and Edwards (2004) argued that the organizational fit between employees' and organizational values is related to determinants such as organizational identity, organizational citizenship behavior, and turnover intention. Past research results have also found a positive relationship between PO fit and job satisfaction (McCulloch and Turban, 2007; Liu et al., 2010; Abdalla et al., 2018; Jehanzeb and Mohanty, 2018). According to the above discussion, we proposed the following four hypotheses.

Hypothesis 1: Person–job fit positively influences job satisfaction.
Hypothesis 2: Person–organization fit positively influences organizational commitment.
Hypothesis 3: Person–job fit positively influences job satisfaction.
Hypothesis 4: Person–job fit positively influences organizational commitment.

Fu and Deshpande (2014) found that there was a positive correlation among job performance, job satisfaction, and organizational commitment in an investigation of 476 insurance practitioners in China. Plentiful research indicated that job satisfaction also affected organizational commitment significantly (Schwepker Jr, 2001; Tsai and Huang, 2008; Malik et al., 2010; Qureshi et al., 2011; Hira and Waqas, 2012). According to the above discussion, we proposed Hypothesis 5 and Hypothesis 6. According to the above discussion, we proposed Hypothesis 5 and Hypothesis 6.

Hypothesis 5: Job satisfaction positively influences organizational commitment.
Hypothesis 6: Job satisfaction positively influences innovative behavior.

The previous empirical evidence pointed out the significantly positive relationship between organizational commitment and innovative behavior in the retail industry from 80 retail executives (Jafri, 2010). Wiener (1982) argues that organizational commitment is an internalized normative force that encourages members of the organization to engage in behavior that is consistent with organizational goals and organizational interests. In summary, this study proposed Hypothesis 7.

Hypothesis 7: Organizational commitment positively influences innovative behavior.

Dereliction of duty means that a worker works in a particular organization for a period of time. After some consideration, he or she deliberately wants to open the organization and loses the original position and the rights and benefits it confers. This is a general attitude and attitude toward dereliction of duty and the search for other job opportunities, often used to explore important predictors of misconduct (Hellman, 1997). Therefore, the employee's turnover intention can explain whether the actual behavior of employee misconduct has occurred. In the discussion of the relevant models of the willingness to work, most of the studies agree that job satisfaction is significantly correlated with the willingness to work (Tsai and Wu, 2010; Park et al., 2014; Jehanzeb and Mohanty, 2018; Shah et al., 2020). Schwepker Jr (2001), through an empirical study of 152 salespeople in 26 companies, points out the negative impact of job satisfaction on employee turnover intention through organizational commitment. Previous research also indicated that job satisfaction and organizational commitment were two important determinants that may affect employee turnover intention (Karsh et al., 2005). Besides, Naz et al. (2020) also found the positive linkage between organizational commitment and employee retention. Therefore, we proposed the last two research hypotheses of this study as follows.

Hypothesis 8: Job satisfaction negatively influences turnover intention.
Hypothesis 9: Organizational commitment negatively influences turnover intention.

**Figure 1:**
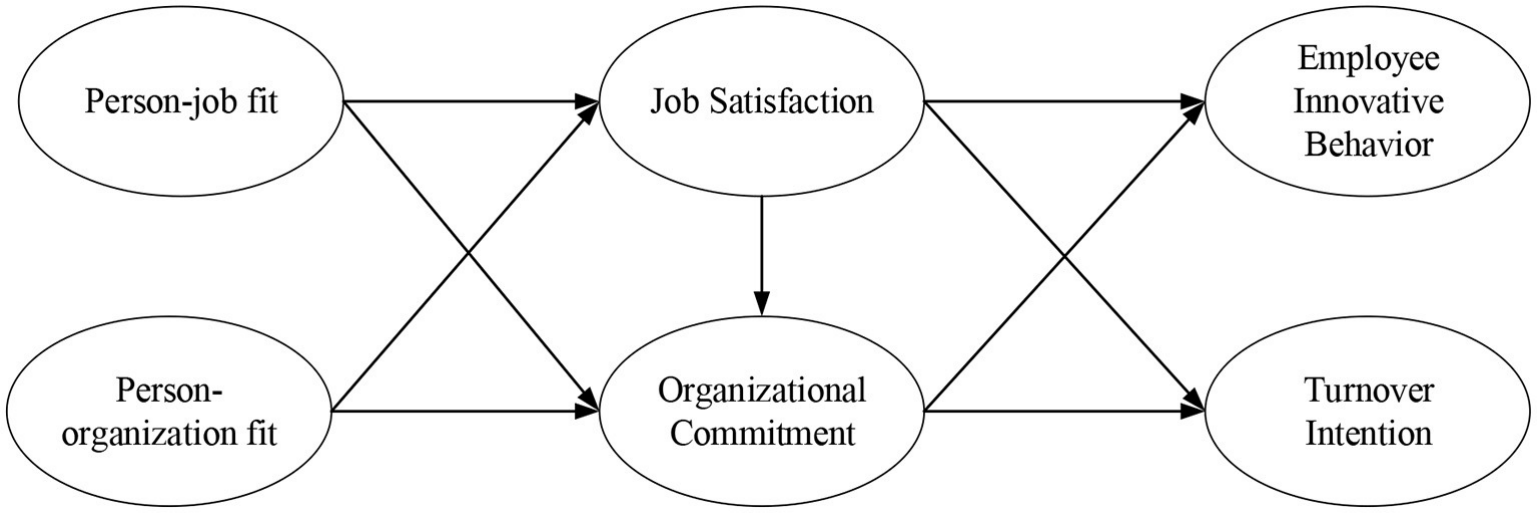
Research model.

Based on the discussion of the aforementioned literature, the relevant research hypotheses (Table 1) and research framework (Figure 1) were proposed in this study.

**Table 1:** Research hypotheses.

Hypothesis 1	Person–job fit has the positive influence on job satisfaction.
Hypothesis 2	Person–job fit has the positive influence on organizational commitment.
Hypothesis 3	Person–organization fit has the positive influence on job satisfaction.
Hypothesis 4	Person–organization fit has the positive influence on organizational commitment.
Hypothesis 5	Job satisfaction has the positive influence on organizational commitment.
Hypothesis 6	Job satisfaction has the positive influence on innovative behavior.
Hypothesis 7	Job satisfaction has the negative influence on turnover intention.
Hypothesis 8	Organizational commitment has the negative influence on innovative behavior.
Hypothesis 9	Organizational commitment has the negative influence on turnover intention.

### Measurement Items and Sample Structure

PO fit developed by Cable and DeRue (2002) was adopted with a three-item scale in this study to examine. An 18-item Multidimensional Person–Job Fit Scale (MPJS) was developed to examine the level of PJ fit. This scale was developed based on Edwards (1991) definition of PJ fit, the research conducted by Cable and DeRue (2002), and the explanatory and confirmatory factor analyses that were performed in accordance with the scale development procedure suggested by Hinkin (1998). The MPJS consists of five constructs, namely, basic needs, self-esteem and attachment, self-realization, job demands, and capabilities, and can be used to accurately understand the fit between employees and their jobs. Six measurement items were developed by Crossman and Abou-Zaki (2003) in this study to measure job satisfaction toward employees. In this study, hypothesis testing was conducted on 697 valid samples from different industrial organizations and different departments in order to improve the external validity of the analysis results. The sample structure of this study is outlined in Table 2.

**Table 2:** Sample structure.

**Category**		**Frequency**	**Percentage (%)**	**Category**		**Frequency**	**Percentage (%)**
**Gender**	Male	469	67.3	Age of company	2–5 years	60	8.6
	Female	228	32.7		6–10 years	152	21.8
Age	<25	15	2.2		>10 years	485	69.6
	26–30	202	29.0	Organizational department	Research and development	352	50.5
	31–35	226	32.4		Procurement	13	1.9
	36–40	152	21.8		Customer service	5	0.7
	41–45	62	8.9		Sales	20	2.9
	>46	40	5.7		Planning	72	10.3
Work experience	<1 year	10	1.4		Administration	65	9.3
	2–3 years	163	23.4		Consulting	4	0.6
	4–6 years	234	33.6		Human resources	43	6.2
	7–9 years	132	18.9		Production/manufacturing	113	16.2
	>10 years	158	22.7		Accounting	10	1.4
Company size	<200 employees	122	17.5	High-tech fields	Electronics	151	22.4
	300–500 employees	264	37.9		Biomedicine	34	4.9
	600–1,000 employees	195	28.0		Aerospace and aeronautics	7	1.0
	>1,000 employees	116	16.6		New materials	53	7.6
Educational level	Diploma and below	82	11.8		High-tech service industry	121	17.4
	Bachelor's degree	445	63.8		Alternative energy and energy conservation technology	178	25.5
	Master's degree	138	19.8		Resource and environmental technology	33	4.7
	Doctoral degree	32	4.6		Reconstruction of traditional industries	116	16.6

### Ethics Statement

About this research, ethical review and approval were not required for this study on human participants in accordance with the local legislation and institutional requirements. Written informed consent from the participants was not required to participate in this study in accordance with the national legislation and the institutional requirements.

## Analysis Results

### Outer Model

There were two stages in the PLS analysis and estimation. In the first stage, reliability and validity analyses were performed on the outer model; in the second stage, estimations and tests were performed on the structural model's path coefficient and explanatory power. These two steps were performed to confirm the reliability and validity of the measurement constructs prior to examining the relationship between each construct (Anderson and Gerbing, 1988). The relevant tests for the outer model mainly covered the internal consistency and convergent, as well as the discriminant validity of each construct.

This study applied Cronbach α value and composite reliability of latent constructs to evaluate the internal consistency of each component. Fornell and Larcker (1981) and Hair et al. (2010) recommended a confidence level of 0.7 or higher to meet the criterion of internal consistency. The values of Cronbach α and composite reliability of each construct were all greater than the recommended values, which indicates good internal consistency in this study.

Convergent validity refers to the degree of convergence between multiple scale items estimating single construct. As shown in Table 3, the average variance extracted and reliability are higher than 0.5 and 0.7, respectively. According to the standard of Fornell and Larcker (1981), the empirical data of this study fulfilled convergent validity.

**Table 3:** Reliability and average variance extracted (AVE) of the outer model.

**Construct**	**Cronbach α**	**Composite reliability**	**AVE**
PJFIT	0.928	0.916	0.688
POFIT	0.845	0.906	0.763
JOBSAT	0.904	0.926	0.675
ORGC	0.908	0.929	0.687
INNO	0.889	0.910	0.530
TURN	0.886	0.911	0.594

Discriminant validity is primarily an examination of the degree of difference between the latent variables in the outer model. The comparison of the factor loadings and cross-loadings for each scale items shows that if the factor loadings for each indicator of its specified construct are higher than its loadings on any other construct, it represents that each construct in this study has reasonable discriminant validity (as shown in Table 4). Therefore, the outer model in this study has good discriminant validity (Hair Jr et al., 2016).

**Table 4:** Standardized factor loadings and cross-loadings of the outer model.

	**INNO**	**ORGC**	**PJFIT**	**POFIT**	**SAT**	**TURN**
INNO1	***0.775***	0.456	0.581	0.523	0.447	−0.323
INNO2	***0.764***	0.409	0.578	0.463	0.379	−0.345
INNO3	***0.742***	0.474	0.582	0.520	0.436	−0.333
INNO4	***0.772***	0.489	0.592	0.522	0.448	−0.368
INNO5	***0.790***	0.460	0.590	0.536	0.443	−0.326
INNO6	***0.682***	0.445	0.535	0.507	0.436	−0.353
INNO7	***0.673***	0.342	0.525	0.425	0.325	−0.236
INNO8	***0.649***	0.260	0.443	0.340	0.240	−0.183
INNO9	***0.690***	0.402	0.499	0.496	0.377	−0.241
JOBSAT1	0.517	0.765	0.707	0.553	***0.846***	−0.709
JOBSAT2	0.432	0.685	0.594	0.491	***0.797***	−0.560
JOBSAT3	0.412	0.666	0.569	0.522	***0.808***	−0.558
JOBSAT4	0.475	0.685	0.610	0.501	***0.792***	−0.557
JOBSAT5	0.406	0.780	0.622	0.540	***0.846***	−0.685
JOBSAT6	0.466	0.789	0.672	0.537	***0.838***	−0.631
ORGC1	0.478	***0.859***	0.673	0.540	0.808	−0.700
ORGC2	0.460	***0.792***	0.583	0.545	0.715	−0.563
ORGC3	0.502	***0.875***	0.669	0.536	0.785	−0.677
ORGC4	0.455	***0.767***	0.545	0.485	0.631	−0.524
ORGC5	0.493	***0.818***	0.634	0.526	0.698	−0.578
ORGC6	0.509	***0.855***	0.674	0.526	0.768	−0.677
PJFIT1	0.526	0.563	***0.721***	0.468	0.566	−0.503
PJFIT10	0.493	0.575	***0.711***	0.526	0.578	−0.424
PJFIT11	0.542	0.486	***0.663***	0.437	0.462	−0.441
PJFIT12	0.542	0.403	***0.628***	0.353	0.390	−0.297
PJFIT13	0.458	0.305	***0.553***	0.291	0.320	−0.203
PJFIT14	0.487	0.407	***0.632***	0.423	0.456	−0.303
PJFIT15	0.430	0.344	***0.539***	0.314	0.337	−0.237
PJFIT16	0.491	0.337	***0.572***	0.338	0.329	−0.188
PJFIT17	0.527	0.366	***0.620***	0.363	0.358	−0.246
PJFIT18	0.507	0.412	***0.612***	0.414	0.403	−0.255
PJFIT2	0.506	0.588	***0.738***	0.500	0.597	−0.498
PJFIT3	0.499	0.588	***0.696***	0.461	0.608	−0.523
PJFIT4	0.545	0.657	***0.760***	0.594	0.694	−0.576
PJFIT5	0.520	0.607	***0.702***	0.575	0.635	−0.521
PJFIT6	0.561	0.606	***0.732***	0.535	0.610	−0.515
PJFIT7	0.532	0.574	***0.730***	0.529	0.577	−0.456
PJFIT8	0.505	0.616	***0.717***	0.548	0.604	−0.483
PJFIT9	0.513	0.603	***0.721***	0.549	0.577	−0.526
POFIT1	0.519	0.501	0.541	***0.845***	0.502	−0.370
POFIT2	0.623	0.577	0.637	***0.878***	0.575	−0.457
POFIT3	0.607	0.580	0.625	***0.897***	0.592	−0.447
TURNO1	−0.259	−0.638	−0.440	−0.397	−0.653	***0.778***
TURNO2	−0.357	−0.636	−0.517	−0.367	−0.609	***0.827***
TURNO3	−0.320	−0.536	−0.436	−0.286	−0.499	***0.734***
TURNO4	−0.317	−0.623	−0.488	−0.358	−0.613	***0.810***
TURNP1	−0.250	−0.549	−0.434	−0.373	−0.587	***0.771***
TURNP2	−0.376	−0.515	−0.479	−0.394	−0.520	***0.740***
TURNP3	−0.421	−0.544	−0.521	−0.462	−0.572	***0.732***

*The bold values are standardized factor loadings and other values are cross loadings for each construct*.

### Inner Model and Mediation Analysis

After discussing the reliability and construct validity of this study, the inner model is then analyzed. In this study, the estimation results of SmartPLS and the path coefficients of the model are used to determine the relationship between each construct. The results of the hypotheses examination are shown in Table 5 and showed that eight of the nine hypothesized relationships in the proposed model were significant.

**Table 5:** Summary of inner model results.

**Hypo**.	**Path direction**	**Standardized path coefficient**	***t* value**	***p* value**	**Result**
H1	PJFIT -> JOBSAT	0.625^***^	13.563	0.000	Supported
H2	PJFIT -> ORGC	0.166^***^	4.187	0.001	Supported
H3	POFIT -> JOBSAT	0.207^***^	5.065	0.004	Supported
H4	POFIT -> ORGC	0.057	1.884	0.237	Not supported
H5	JOBSAT -> ORGC	0.725^***^	19.827	0.000	Supported
H6	JOBSAT -> INNO	0.156^*^	2.234	0.026	Supported
H7	JOBSAT -> TURN	−0.408^***^	6.629	0.000	Supported
H8	ORGC -> INNO	0.444^***^	6.339	0.000	Supported
H9	ORGC -> TURN	−0.390^***^	6.014	0.001	Supported

*Note:*

* *p < 0.05*,

* *p < 0.01*,

*** *p < 0.001*.

This study applied the method of bootstrapping to estimate the confidence intervals of the mediation effect to prevent the asymmetric indirect path product coefficient (Williams and MacKinnon, 2008; Hayes, 2009). If the confidence interval of the bootstrap does not contain 0, then an indirect effect exists. Incidentally, if an intermediary effect is not significant for one of the paths, the intermediary effect associated with that path is not included in the analysis (i.e., POFIT -> ORGC) (Table 6).

**Table 6:** Mediation effect examination.

**Mediation path**	**Path coefficient (O)**	**Standard deviation (Stdev)**	***T* statistics (|O|STDEV|)**	**CI lower bound 2.5%**	**CI upper bound 97.5%**
PJFIT -> ORGC -> INNO	0.074^**^	0.026	2.876	0.033	0.135
PJFIT -> SAT -> ORGC -> INNO	0.201^***^	0.030	6.624	0.138	0.260
SAT -> ORGC -> INNO	0.322^***^	0.046	6.992	0.222	0.411
PJFIT -> SAT -> INNO	0.097^*^	0.046	2.141	0.012	0.194
POFIT -> SAT -> INNO	0.032^*^	0.016	2.019	0.005	0.068
PJFIT -> SAT -> ORGC	0.454^***^	0.035	13.053	0.382	0.520
POFIT -> SAT -> ORGC	0.150^***^	0.030	5.061	0.094	0.214
PJFIT -> ORGC -> TURN	−0.065^**^	0.019	3.321	−0.110	−0.032
PJFIT -> SAT -> ORGC -> TURN	−0.177^***^	0.030	5.975	−0.233	−0.117
SAT -> ORGC -> TURN	−0.283^***^	0.047	6.034	−0.371	−0.191
POFIT -> SAT -> ORGC -> TURN	−0.059^***^	0.016	3.701	−0.093	−0.032
PJFIT -> SAT -> TURN	−0.255^***^	0.044	5.818	−0.346	−0.171
POFIT -> SAT -> TURN	−0.085^***^	0.020	4.274	−0.124	−0.049

* *p < 0.05*,

** *p < 0.01*,

*** *p < 0.001*.

## Discussion and Conclusion

With the rapid development of knowledge-based economies and technological upgrades, organizational environments have brought immense risks and competition pressures, which implies that employees are the main pushing forces for innovation. The current process of recruiting, assessing, and developing talents should not only consider the match between an individual's capabilities and the job requirements, instead, and more importantly, it should also implement effective approaches to measure the fit between individual and organizational characteristics. Therefore, the relevant empirical results achieved in this study are expected to complement the gaps between relevant fields of research. Our findings bear significant meanings in the current time, and the COVID-19 pandemic has affected the society seriously, and many workers were laid off consequently (Heinonen and Strandvik, 2020).

As innovation plays a strategic role in transformation, every organization should pay attention to innovative measures that can continuously enhance their core advantages. As competitors are always ready to imitate, organizations must constantly create new knowledge and engage in innovative behavior (Tsai, 2011). According to Dobni (2008), innovation is a company's long-term competitive advantage. Therefore, managers should understand the importance of innovation and instill innovation in their employees.

In addition, because of social, political, and economical changes, as well as immense international competition, internal reforms frequently occur within organizations, such as shifts in strategies, structural adjustments, and systematic innovations. As organizational functions and task execution become increasingly complex, the replacement of individuals with work teams as the basic organizational structure unit has become crucial for companies to achieve their visions (Mathieu et al., 2008), because team strength can be built from each individual's capabilities and attributes to generate faster responses, task-oriented efforts, and organizational productivity (Montoya-Weiss et al., 2001).

The important findings of this study are summarized as follows. First, organizations should implement tests during the recruitment process to check the individual values of job seekers, so that employees with high PO value fit can be selected, which is beneficial for enhancing employees' job satisfaction and organization performance. Next, PJ fit and PO fit were found to be closely related to employees' job satisfaction and turnover intention. In addition to implementing training centered on role familiarization and skills building, the process of socialization should also emphasize organizational culture to enhance interactions between individual and organizational culture, enhance employees' understanding of and identification with organizational values, and increase the fit between individual and organizational values. Not only can this enhance the job competence of employees, but may also increase employees' identification with organizations, thereby boosting their morale and the stability of work teams. Furthermore, managers should consider approaches such as scheduling regular meetings to achieve value identification with employees. Value fit should also be considered as an important indicator for management performance. Finally, continuous self-assessment of PJ fit and PO fit among organizational members is beneficial for the planning of individual career prospects. If an individual's PJ fit is weak but they identify with organizational values, they should consider transferring to other positions within the same organization, whereas if an individual is competent in a certain job but fails to adapt to the organization, they should consider searching for similar jobs in other organizations. Therefore, job seekers should perform a complete self-assessment, while organizations should develop assessment tools by integrating PJ fit and PO fit to assess employees' level of fit and, accordingly, implement various management practices. This approach allows an organization to effectively supervise and reward employees and also enables it to maintain stable and promising work teams that enhance the competitive advantage of organizations.

Resilience, bounce-back from the failure, matters most in innovative sectors (Liu and Liang, 2015). Innovative sectors, such as high-tech industries, are facing intense competition from global markets and are extremely vulnerable in a volatile business environment (Liu and Liang, 2015). Therefore, the balance between the innovative behavior and resilience should be carefully made in order to achieve the sustainable development of these companies (Todt et al., 2018).

Our findings investigated the relations between PE fit and employees' innovative behaviors. Previous empirical studies have shown that PE fit (PJ fit and PO fit) has statistically significant effects on job satisfaction, organizational commitment, and retention intentions toward employees in the enterprises. A high PJ fit and PO fit not only can increase individual work performance, but also can have distinct effects on long-term organizational outcomes, which closes the gap between individual and organizational values, goals, and characteristics, thereby increasing their compatibilities and creating a harmonious organizational atmosphere that promotes organizational development. Hence, relevant studies on PE fit (PJ fit and PO fit) have important theoretical and practical significance for enhancing organizational performance and for constructing harmonious organizations. Thus, results of these studies can provide a new recruitment model for companies to attract new talents while retaining key talents, in addition to providing relevant theoretical and supporting methods for research on personnel recruitment. The level of PJ fit and PO fit has direct effects on the rational utilization of a company's resources and its overall allocation effectiveness, as it is a crucial factor for determining a company's sustainable and stable development. The current process of recruiting, assessing, and developing talents should not only consider the match between an individual's capabilities and the job requirements, but most importantly, also should involve the implementation of effective approaches to evaluate the fit between individual and organizational characteristics. Hence, this study aimed to provide beneficial theoretical support for human resource management by highlighting a novel management concept and strategies for organizational sustainable development.

Even though this study has strived to meet the rigor of social studies research standards, the following limitations have to be considered. First, this research was based on individuals, whereas a complete company should include individuals, departments, and organizations. Activities involving organizational innovation, turnover intentions, and human resource management have many themes that cross over other levels, but as most studies have been based on a single level, future research may produce more accurate results by integrating macro– and micro–crossover-level theories and research. Next, the cross-sectional research approach was adopted in this study, which limits the extent to which the relationships between outcome variables can be inferred. Finally, factors affecting employees' and organizations' innovative behavior were not restricted to those considered in this study. For instance, Germain et al. (2001) suggested that a company's performance level can be determined by understanding the willingness of employees to share their knowledge with other organizational members. Bock and Kim (2002) also revealed that knowledge sharing cannot be properly promoted simply through cash remuneration alone. Hence, it is suggested that the number of variables may be increased in future studies for the sake of achieving a higher degree of accuracy.

## Data Availability Statement

The original contributions presented in the study are included in the article/supplementary material, further inquiries can be directed to the corresponding author/s.

## Ethics Statement

Ethical review and approval were not required for this study on human participants in accordance with the local legislation and institutional requirements. Written informed consent from the participants was not required to participate in this study in accordance with the national legislation and the institutional requirements.

## Author Contributions

YT conceived and designed the research, wrote, and revised the manuscript. Y-FS, Y-JC, and YM gave guidance throughout the whole research process. All authors contributed to the article and approved the submitted version.

## Conflict of Interest

The authors declare that the research was conducted in the absence of any commercial or financial relationships that could be construed as a potential conflict of interest.
